# Machine learning to improve predictive performance of prehospital early warning scores

**DOI:** 10.1038/s41598-025-08247-0

**Published:** 2025-07-01

**Authors:** Logan Morgan Ward, Tim Alex Lindskou, Mads Lause Mogensen, Erika Frischknecht Christensen, Morten Breinholt Søvsø

**Affiliations:** 1 Centre for Prehospital and Emergency Research and Danish Centre for Health Services Research, Aalborg University Hospital and Department of Clinical Medicine, Aalborg University, Selma Lagerløfs Vej 249, 9260 Gistrup, Denmark; 2 Treat Systems ApS, Hasserisvej 125, 9000 Aalborg, Denmark; 3 Emergency Medical Services, North Denmark Region, Hjulmagervej 20, 9000 Aalborg, Denmark

**Keywords:** Early warning score, Emergency medical services, Machine learning, Vital signs, Mortality, Diagnosis, Preclinical research, Risk factors

## Abstract

Early warning scores are used to assess acute patients’ risk of being in a critical situation, allowing for early appropriate treatment, avoiding critical outcomes. The early warning scores use changes in vital signs to provide an assessment, however they tend to identify a considerable number of false positive cases, especially among prehospital patients. We investigated the development and validation of predictive scores based on machine learning models among patients (aged ≥ 18 years) who used ambulances in the North Denmark Region from July 1, 2016, to December 31, 2020. The machine learning models were compared to standard early warning scores (NEWS2 and DEPT), on 7- and 30-day mortality and intensive care admission. The cohort of 219,323 patients was split into development (n = 175,458 (80%)) and validation (n = 43,865 (20%)) datasets to respectively develop and test the machine learning models. These models were logistic regression, random forest, Bayesian networks, and gradient boosting. The machine learning models outperformed NEWS2 and DEPT, with fewer false positives, reducing the number of patients needed to screen by nearly half, for 7 day mortality. This has the potential to reduce both under- and over-triage, improving the precision of the triage among prehospital patients.

## Introduction

Prediction of serious adverse outcomes is essential in emergency care, and the focus of much research. Early warning scores (EWS) are used to assess acute patients’ risk of critical deterioration by identifying early changes in vital signs and adjust treatment, avoiding critical outcomes. To maximize the potential benefit on patient outcome, EWS should be used as early as possible in the healthcare pathway, preferably already among patients treated by emergency medical services (EMS) in the prehospital phase^1,2^.

Previous research indicates that EWS only have moderate predictive performance in the prehospital setting. However, despite some large studies^1,3,4^, many are based on only few patients, and often include a select group of potentially critically ill or injured patient^2,5^. We previously assessed the ability of EWS to predict mortality and intensive care unit stay, in a cohort of 219,323 unselected adult ambulance patients, also finding only moderate performance for the included EWS. Previous studies have mainly focused on EWS’ sensitivity to avoid under-triage, i.e. overlooking critically ill patients (false negatives)^6^. Despite this, many false negatives are found, including in our own study. However, comparatively less focus has been placed on over-triage caused by the low specificity of EWS leading to a high number of false positive cases, thereby potentially giving rise to too many ‘false alarms’ by alerting of advanced medical teams in the emergency department (ED)^7^. The high pressure on the entire emergency system, prehospital and in hospital, emphasizes the importance triage systems of greater precision to minimize both under- and over-triage.

With the emergence of electronic prehospital medical records, including vital signs, there are opportunities to make use of greater computational abilities for ML or advanced statistical models. Use of ML models has been shown to improve the predictive performance of EWS relative to standard clinical scores^8–11^. However, despite the development of many risk models, few to none have been implemented in clinical practice. One of the barriers to clinical use is the “black-box” nature of some ML models.

This article is a continuation of our previous work, with the aim to investigate the use of machine learning (ML) models to improve the precision of EWS in predicting short term mortality in an unselected prehospital patient population. In addition to predictive performance, we present examples of model explanation, and how these can be used to improve understanding of the underlying workings of the ML models.

## Methods

### Design

Prognostic population-based development and validation study based on a historic consecutive cohort of adult ambulance patients. Findings reported in accordance with the TRIPOD statement^12^.

### Setting

The North Denmark Region counts 550,000 inhabitants.

Every Danish citizen has a unique civil registration number used in health care contacts and allowing for registry linkage. The citizen’s sex is defined with this number (even or uneven), which indicates either female or male. It is possible to request an ambulance either by calling the national emergency number, or via primary care (general practitioners or out-of-hours service) for less severe conditions. All prehospital vehicles used the same prehospital medical record containing, amongst others, measured vital signs which are automatically transferred from the ambulance to the medical record.

### Participants

Patients ≥ 18 years using ambulance services in the North Denmark Region from July 1, 2016, to December 31, 2020, were included. Patients with missing civil registration number, time of death prior to record-creation date, no vital sign measurements, with no unique medical record linkage or where diagnoses concerning death were received at hospital arrival were excluded. Both patients in ambulances requested by calling the emergency number or requested by general practitioners were included. Patient transfers, referrals or other prehospital contact without a need for observation and treatments, were not included. The study cohort is previously described^7^.

### Ethics

Informed consent waiver: The need for informed consent was waived by The Danish Patient Safety Authority.

According to Danish legislation, informed patient consent for the handover of medical records is required. However, this requirement can under certain circumstances be waived, for specific health science, as was the case in the current study. The Danish Patient Safety Authority approved the handover of the prehospital medical records (reference ID 2021–012,621). No further approval (e.g. by ethics committee) is required for registry-based studies, such as the current study. These procedures are in accordance with Danish law, and all methods were carried out in accordance with relevant guidelines and regulations.

### Variables and data sources

Prehospital vital signs (*heart rate, systolic, diastolic, and mean arterial blood pressure, blood oxygen saturation (SpO2), respiratory rate, blood glucose level, temperature, pain (visual analogue scale score), and Glasgow Coma Scale score*) were retrieved from the prehospital medical record, and ambulance logistic data from the logistic systems. Sex, age, and possible death date were collected from the Danish Civil Registration System. Hospital data (ED contact, admission and discharge dates, admitting department) and diagnoses according to the International Statistical Classification of Diseases and Related Health Problems, Tenth Revision (ICD-10) were collected from the regional patient administrative system. All data sources were linked by civil registration numbers.

### Outcomes

The primary outcome was 7-day mortality. Secondary outcomes were 30-day mortality and ICU admission.

### Comparators

To compare with current clinical practice, two EWS: National Early Warning Score 2 (NEWS2; score range, 0–20) and Danish Emergency Process Triage (DEPT; score range, 1–4), were included^13,14^. These EWS represent different triage techniques: NEWS2 is additive and bases actions on the combined score across vital signs while DEPT categorizes patients into one of four triage levels using the single worst vital sign. Calculation methods for the EWS and performance for the cohort are reported in previous work^7^. Simple logistic regression models combining each EWS with age were also constructed.

### Model development

The classification models were based on four representative ML algorithms: gradient boosting machine (GB), random forest (RF), logistic regression (LR), and Bayesian network (BN).

The dataset was partitioned into training (80%) and test (20%) datasets prior to feature selection, using stratified random sampling.

Features were added for statistical and temporal summaries. A full description is included in the Supplementary Method Material online. Features were selected on significant univariate predictive ability. Three feature selection strategies were used to provide an indication of how the models perform given increasingly restrictive sets of data. Set 1 included summary statistics for temporal variables (heart rate, systolic, diastolic, and mean arterial blood pressure, blood oxygen saturation, respiratory rate, blood glucose level, temperature, pain, and Glasgow Coma Scale score) across the full episode. Set 2 comprised variables in Set 1 plus patient age. Set 3 comprised variables in Set 2 plus sex, comorbidities, and the source of the ambulance (emergency call/GP requested). Vital signs were summarized at the episode level as first, last, minimum, LQ, median, UQ, maximum and change from first.

Each model type handles missing data differently. GB and BN models handle missing data inherently. For RF, extreme values were imputed to indicate missingness. For LR, a simple median imputation was used, as it required the fewest assumptions about the nature of the missingness. Univariate predictive performance and missingness can be seen in the Supplementary Materials Fig. s1.

GB, RF, and LR algorithms were tuned using a tenfold cross-validation scheme with randomized grid-search to select optimal hyperparameters for the target variable 7-day mortality. Following hyperparameter selection, each model was then retrained using the complete training dataset. All model training was performed in python using the scikit-learn package.

The BN requires two elements to be specified or learned: the model’s graphical structure and the conditional probability tables. The graph was specified manually, based on clinical knowledge and prior experience. The model structure and assumptions are shown in Supplementary Materials online. The conditional probability tables were learned using the Expectation–Maximization (EM-) learning algorithm implemented in the Hugin Expert software.

### Statistical analysis

Descriptive statistics are presented as number (percent) for categorical variables and median and inter-quartile range (IQR) for non-normally distributed continuous variables.

Differences in distributions of categorical and continuous variables were assessed using the Chi-squared test and Wilcoxon rank-sum test, respectively.

Predictive performance was calculated as the area under the receiver operating characteristic curve (AUROC) and the area under the precision recall curve (AUPRC). Calibration was assessed visually and by calculating the Brier loss. Calibration curves were plotted for observed vs. predicted risk, across ten deciles. AUROC, AUPRC and Brier loss were calculated using sci-kit learn.

Performance measures (AUROC, AUPRC, sensitivity, specificity, positive predictive value (PPV), negative predictive value (NPV)) are presented with 95% confidence intervals (95% CI). Confidence intervals were calculated using 1,000 bootstrap resamples. Differences in AUROC and AUPRC were computed via bootstrapping.

Point-estimates of performance (sensitivity, specificity, PPV, and NPV) were calculated for standard thresholds for the clinical scores. Standard thresholds were taken as NEWS2 = 5; the threshold suggested for the patient to be examined by a doctor, and DEPT = 3 (orange); which is the threshold for high priority treatment^8,9^. ML model thresholds were chosen to match the sensitivity of NEWS2 = 5 and DEPT = 3.

Data manipulation and calculation of statistical tests were carried out using python (scikit-learn v1.3.2, SciPy v1.11.3 in python version 3.10.12).

### Stratified/Subgroup analysis

Stratified analyses were performed grouping patients by diagnosis category (main chapter of ICD-10 for the first diagnosis received at hospital), age group, and sex. If the first diagnosis received at the hospital was non-specific (ICD-10 main chapter 18 “symptoms and signs” or chapter 21 “other factors”), we reassigned the patient an organ- or pathology-specific diagnosis if this was available for the hospitalization.

### Sensitivity analysis

To determine the effect of requiring a prediction to be made prior to the end of ambulance transport, analyses were repeated using only data available up to a series of fixed intervals (5, 10, 15, 20, 25, 30 min) following the initial measurement.

To investigate the impact of patients who did not survive day 1, the analysis was repeated for 2–7 day and 2–30-day mortality, excluding all patients who died on day 0–1.

### Model explanations

For a set of example cases, model predictions are shown together with an explanation of the impact of each input variable on the output. Details of feature impact assessment for each model are shown in the Supplementary Materials tables s1, s2, s3, s4.

## Results

### Patient population

219,323 medical records were included for 107,569 unique patients. Details of inclusion/exclusion are shown in Supplementary Materials Fig. S2. Descriptive statistics for included patients are shown in Table 1.

**Table 1:** Descriptive statistics.

Characteristic	Patients, No. (%) unless otherwise stated		
	Total cohort (N = 219 323)	Deceased day 7 (N = 9 344)	Alive day 7 (N = 209 979)
Age, median (IQR), y	69 (52–80)	79 (70–87)	69 (51–80)
Sex			
Female	104 699 (47.7)	4 150 (44.4)	100 549 (47.9)
Male	114 624 (52.3)	5 194 (55.6)	109 430 (52.1)
Called emergency number	119 992 (54.7)	4707 (50.4)	115 285 (54.9)
Charlson comorbidity index, points, median (IQR)	0 (0–1)	1 (0–2)	0 (0–1)
Acute severity, points			
NEWS2, median (IQR)	3 (1–5)	5 (3–8)	3 (1–4)
DEPT, median (IQR)	2 (1–3)	4 (3–4)	2 (1–3)
Admission			
Hospital	198 264 (90.4)	7445 (79.7)	190 819 (90.9)
ICU admission	5 044 (2.3)	661 (7.1)	4 383 (2.1)
Mortality, crude			
1-day	4 119 (1.9)	4 119 (44.1)	0 (0)
7-day	9 334 (4.3)	9 344 (100)	0 (0)
30-day	18 650 (8.5)	9 344 (100)	9306 (4.4)

### Discriminatory performance

The main analysis considers models trained with feature set 2 (vital signs and age), to provide a realistic comparison with the status quo, using data that are currently available in the ambulance at the time of triage. Full results for all feature sets are included in the Supplementary Materials Table S4.

**Fig. 1:**
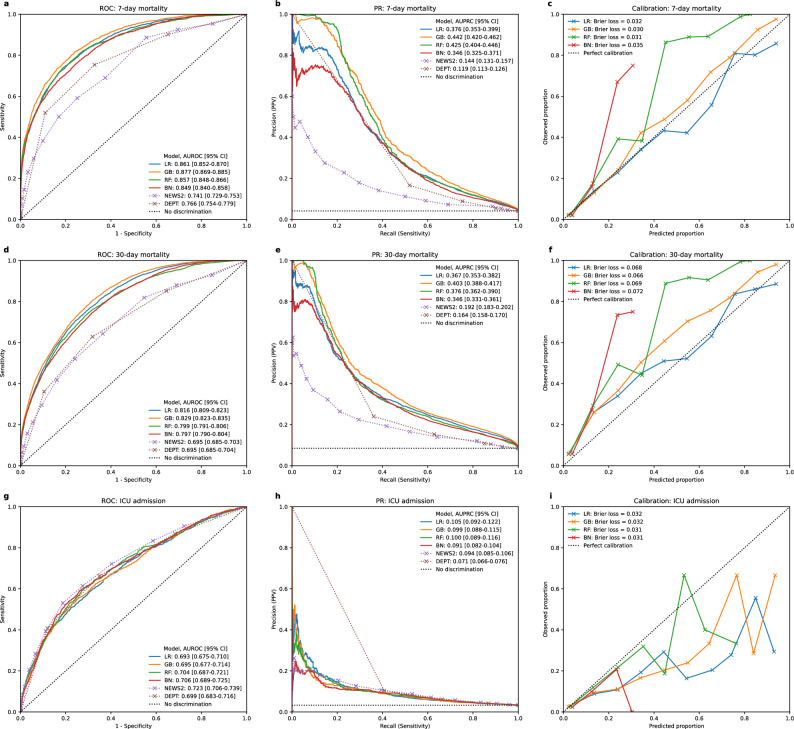
Test set performance and calibration of ML models. *ROC* Receiver Operating Characteristic, *PR* Precision-Recall.

Figure 1 shows overall discriminatory performance and calibration for the ML models. All ML models had higher AUROC and AUPRC compared to the two clinical EWS. GB and LR were well calibrated for the primary outcome, while RF and BN tended to underpredict.

### Performance at threshold values

As a representative use case, thresholds were selected for the most performant model (GB) and for BN to match the sensitivity of NEWS2 = 5 and DEPT = 3. Performance measures are reported for the primary outcome, 7-day mortality. Matching sensitivity, GB and BN had significantly fewer false positives, reducing the degree of over-triage compared to NEWS2 and DEPT. However, use of a single threshold highlights the remaining issue of under-triage, where 25–40% of patients meeting the 7-day mortality outcome are missed by the NEWS2 = 5 and DEPT = 3 cut-offs (Table 2).

**Table 2:** GB performance at sensitivity-matched thresholds, primary outcome: 7-day mortality, test set (N = 43 865).

	High risk matching NEWS2			High risk matching DEPT		
	NEWS2 = 5 (N = 43 811)	GB	BN	DEPT = 3 (N = 43 777)	GB	BN
Alarms, n (%)	11 627 (26.5)	4514 (10.3)	5250 (12.0)	15 087 (34.5)	9234 (21.1)	11,175 (25.5)
True positives, n	1060 (9.1)	1089 (24.1)	1060 (20.2)	1332 (8.8)	1381 (15.0)	1359 (12.2)
False positives, n	10 567 (90.9)	3425 (75.9)	4190 (79.8)	13 755 (91.2)	7853 (85.0)	9816 (87.8)
False alarms per true alarm (95% CI)	10.0 (9.5–10.7)	3.1 (2.9–3.4)	4.0 (3.7–4.2)	10.3 (9.8–10.8)	5.7 (5.4–6.0)	7.2 (6.8–7.7)
No alarm, n (%)	32 184 (73.5)	39 351 (89.7)	38 615 (88.0)	28 690 (65.5)	34 631 (78.9)	32 690 (74.5)
True negatives	31,451 (97.7)	38 618 (98.1)	37,853 (98.0)	28,254 (98.5)	34,190 (98.7)	32,254 (98.7)
False negatives	733 (2.3)	733 (1.9)	762 (2.0)	436 (1.5)	441 (1.3)	436 (1.3)
Performance measures, median (95% CI)						
Sensitivity*	0.591 (0.566–0.611)	0.598 (0.576–0.619)	0.582 (0.559–0.603)	0.753 (0.733–0.769)	0.758 (0.740–0.778)	0.746 (0.726–0.767)
Specificity	0.749 (0.745–0.753)	0.919 (0.916–0.921)	0.900 (0.897–0.903)	0.673 (0.669–0.676)	0.813 (0.810–0.817)	0.767 (0.763–0.771)
Positive predictive value	0.091 (0.085–0.095)	0.241 (0.228–0.255)	0.202 (0.191–0.213)	0.088 (0.085–0.093)	0.150 (0.142–0.156)	0.122 (0.115–0.128)
Negative predictive value	0.977 (0.976–0.979)	0.981 (0.980–0.983)	0.980 (0.979–0.982)	0.985 (0.983–0.986)	0.987 (0.986–0.988)	0.986 (0.985–0.987)

*In each case, GB and BN thresholds were chosen to match the sensitivity of the clinical EWS.

Predictive performance varied according to the diagnoses given in hospital, with higher performance for patients diagnosed with circulatory diseases and diseases of the nervous system (Supplementary Materials online Figs. S3 and S4).

### Effect of individual features

**Fig. 2:**
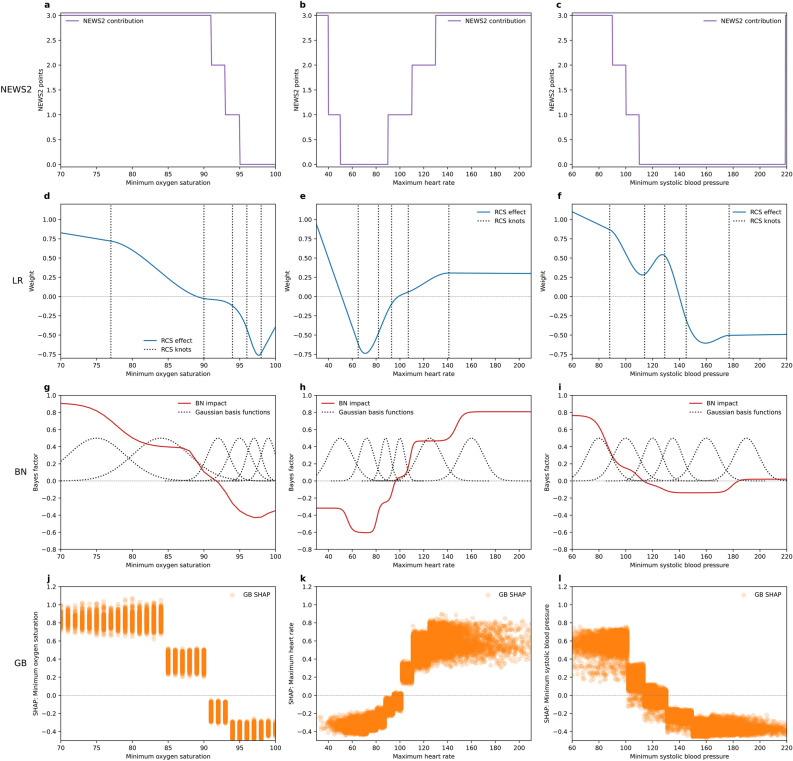
Model impact: Left, top to bottom: effect of minimum oxygen saturation on model probability of 7-day mortality (or score) for NEWS2, LR, BN and GB. Centre, top to bottom: effect of maximum heart rate. Right, top to bottom: effect of minimum systolic blood pressure. *RCS* restricted cubic splines.

Figure 2 contrasts the effect of three single features (oxygen saturation, heart rate and systolic blood pressure) on the model output for the different modelling techniques. In each case, larger impact signifies stronger association with the 7-day mortality outcome, while negative impact signifies protective effect—association with 7-day survival. Each model shows a similar pattern, with small differences in threshold locations and impact strength.

### Model explanations

**Fig. 3:**
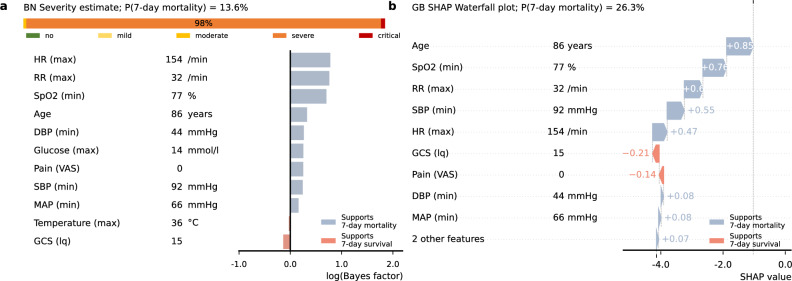
Figure explanations for example cases. The left panels (**A**) show the BN output (probability of 7-day mortality, estimation of severity) and explanations of parameter impact. The impact of each observation is shown with measurements supporting prediction of 7-day mortality represented with blue bars in the positive axis direction and those supporting survival with red bars in the opposite direction. Similarly, the right panel (**B**) shows the explanations for GB as a SHAP waterfall plot. *HR* heart rate, *RR* respiratory rate, *DBP* diastolic blood pressure, *Glucose* blood glucose level, *SBP* systolic blood pressure, *MAP* mean arterial blood pressure, *GCS* Glasgow Coma Scale score.

Figure 3 presents a case, typical for a patient with COPD exacerbation presenting to emergency care, elderly, awake patient with high heart rate and respiratory rate, and low oxygen saturation, showing deviation between the GB and BN models. For each model, observations contributing to higher estimated severity are shown in blue bars, with observations acting to reduce estimated severity shown in red bars. Both models behave similarly in terms of the effect of individual parameters, with some variation in weighting. The BN model is most impacted by heart rate and respiratory rate (ranks 5, 3 in GB) while the GB model is most impacted by age and SpO2 (ranks 4, 3 in BN), and in both cases GCS 15 points towards higher chance for survival. Presenting additional information showing how the model arrived at its conclusion may help to build trust in their use.

### Supplemental subgroup and sensitivity analyses

The ML models maintained their advantage over the EWS for all subgroups. The most performant model (GB) had AUROC 0.839 (95% CI 0.830–0.848) at the 5-min horizon compared with 0.877 (95% CI 0.869–0.885) for the end of episode data. All models showed reduced performance when excluding patients who died on day 1. Significant deviations in performance were seen across diagnosis-, age-, and sex- subgroup analyses (Supplementary Material online Figs. S5–S8).

## Discussion

This study’s purpose was to investigate the use of ML models to improve precision of EWS in predicting short term mortality in an unselected prehospital population. The ML models performed significantly better than the standard clinical EWS (NEWS2, DEPT), with fewer false positives (reduced over-triage) at sensitivity-matched thresholds.

Age was an important predictor in all ML models. When age was added to NEWS2 and DEPT in regression models, performance was increased in line with other studies in the field^15^, but was still below the level of the ML models.

Studies comparing different methods of ML models to EWS have found results in accordance with ours, including three Nordic studies^7,10,11^. Where tested, the ML models outperformed NEWS for prediction of patient admission (1/1 study), ICU admission (2/3 studies), and short-term mortality (1–2 days mortality) (2/3 studies). A similar study from Japan did not compare models with existing EWS but used an approach of testing multiple feature sets to predict patient admission^9^. The most performant model was GB using age, sex, chief complaint, and vital signs with AUROC of 0.818, sensitivity of 0.744, and specificity of 0.745. Compared to our study, these included fewer patients and had greater selection bias^8–11^. They did also not investigate potential operating points for clinical implementation or methods for model explainability to address user trust.

In our study, performance of all models increased with time due to more available data, yet the models showed good performance with the first measurements of vital signs within the first five minutes, which is of importance for the initial decision made by the EMS professionals on-scene. Moreover, model performance improved within the first 20 min, corresponding to typical on-scene times for the EMS^16^. These models should be tested in prospective studies and given that the models performance persisted it should be investigated whether this improves patient outcome and/or decrease the number of alarms received by the team at the hospital in false positive cases in randomized controlled studies.

Significant deviations in performance were seen across diagnosis-, age-, and sex- subgroup analyses. However, the pattern of deviation was similar across both NEWS2, DEPT and the age-adjusted models. For most subgroups, the predictive advantage of the ML models remained. The use of ML does not appear to introduce additional bias relative to the existing EWS. However, it does highlight the difficulty of universal triage models for unselected patient groups.

For the dataset in this study, higher degrees of missingness are correlated with higher mortality, which has also been seen in other studies^7,17,18^. In emergency situations, missing data may be associated with lack of clinical relevance, leading to assuming missing = normal for triage. However, in ambulance cases, the reasons for missingness may include both lack of clinical relevance or a focus on resuscitation and live-saving care over making diagnostic measurements. Among pediatric patients requiring ambulance services, missing data has been reported as being associated both with mild- and with more severe disease^19^. An alternative approach would be to exclude patients without complete data. However, this would result in a large selection bias, as in the study of Pirneskoski et al., where the initial population of 589,397 adult patients was reduced to 26,458 patients after excluding those with incomplete data^11^. The excluded patients with no measured vital signs in the current study may represent any of the mentioned above cases, or indeed other issued. As such, these patients represent an unknown, as their medical situation cannot be determined and thereby may skew the results in all directions.

The true impact of missing data varies according to the model used. LR requires complete data, which were provided by an imputation scheme, imputing the median measured value across the training data. Although the resulting score was reasonably performant, this technique does not lead to an intuitive explainable model in the real-world context of missing data. GB handles missing data at the training stage by assigning these to the most similar branch of the tree where a split is defined. RF does not inherently handle missing data. In this study, missing data were tagged as extreme negative values, allowing splits to be made where these were informative. GB and RF performance may suffer when trying to generalize to other settings if they have different patterns of missingness. In contrast, BN handles missing data inherently through its prior distributions, effectively acting as a knowledge base.

Clinical decision support systems need to be trustworthy to gain the full benefit of their relative improvements in performance. Model explainability is an important factor in developing this trust^20,21^. Explainability may be inherent to the model, due to its interpretability e.g. contribution of variables in simple regression models, the path traversing a decision tree, variable impact in Bayesian networks; or may be achieved through computational methods. A particular advantage of the BN model is the ability to read off probabilities for any node in the network. This allows the same model to provide e.g. an estimation of acute severity and/or the probability of multiple outcomes with the same model.

There has been significant progress in explainability for non-interpretable models such as GB and RF, such as the use of Shapley additive explanations (SHAP) or local interpretable model-agnostic explanation (LIME)^22,23^. The SHAP examples provided in this study show a simple view of the contributions of each variable to the prediction for each example case.

In prehospital triage, there is a significant degree of both under-triage where potentially critical cases are missed, and over-triage where mild or moderate cases are (perhaps) unnecessarily triaged as critical. In this study, we used 7-day mortality as a proxy for risk, despite it being distal to the time of measurement. As such, several intermediate effectors apart from prehospital vital signs may influence the patient’s outcome. Likewise, the use of ICU may be highly influenced by local resources.

In the current study, particular for high-risk patients, we would expect some level of “over-triage”.

The current study assessed to use of the ML-model scores as screening tools and therefore chose to include patients with subsequent encounters within the follow-up period. As such the second encounter could in some cases be dependent in some degree on events associated with the first encounter. This emphasizes the limitations when using mortality as a proxy for acute severity. In contrast, using only the first encounter for a patient would likely result in an underestimation, whereas only the last would provide an overestimation. These limitations are also part of the reason for the choice of 7-day mortality as the primary outcome.

EWS based on ML-models have potential to significantly improve the precision of prehospital triage compared with existing EWS in daily clinical use. Implementing the ML-models in the prehospital medical record would provide real-time calculations of the scores. This will allow prehospital personnel to initiate early treatment in high-risk patients, and likewise safely treat and release those in low risk. However, prospective research is needed to collect data on real-world effectiveness and viability of these methods.

## Supplementary Information

Supplementary Information.

## Data Availability

The datasets used and/or analysed during the current study available from the corresponding author on reasonable request.
