# Supplementary material for: Machine learning to improve predictive performance of prehospital early warning scores
Source: Sci Rep. 2025 Jul 1;15:21459. doi: 10.1038/s41598-025-08247-0 (PMC12215262; doi:10.1038/s41598-025-08247-0)
Supplement: Supplementary file 1 — Supplementary Information. [file 41598_2025_8247_MOESM1_ESM.pdf]

Supplemental material

This supplemental material contains tables and figures with additional information regarding the model training and selection, the primary analysis and subgroup and sensitivity analyses.

Contents

Supplemental Methods

Additional description of ML models and training and evaluation methods.

Supplemental Results

Additional details of subgroup and sensitivity analyses.

Tables

Table s1 Descriptive statistics for the cohort stratified by train/test

Table s2 Predictive performance for candidate variables in the training dataset for the primary outcome 7-day mortality

Table s3 Hyperparameter arrays used for model selection and chosen hyperparameters selected by best cross-validation performance

Table s4 Performance of candidate models on the train and test sets

Figures

Figure s1 Univariate predictive performance and missingness

Figure s2 Inclusion flowchart, reproduced from[1]

Figure s3 Subgroup analysis stratified by ICD-10 diagnosis group ***(AUROC)***

Figure s4 Subgroup analysis stratified by ICD-10 diagnosis group ***(AUPRC)***

Figure s5 Subgroup analysis stratified by age

Figure s6 Subgroup analysis stratified by sex

Figure s7 Sensitivity analysis comparing model performance using data collected up to a set of time horizons

Figure s8 Sensitivity analysis comparing full dataset performance to performance where patients who died on day 1 are excluded

Supplemental References

Methods

Feature Engineering and Selection

The original dataset consisted of static variables (e.g. age, comorbidities, emergency call type) and temporal variables with timestamped measurements, with multiple measurements of each variable available for a given patient (e.g. vital signs). For the temporal variables, at each measurement timepoint, running statistical summaries were calculated. The following summary statistics were used: first, last, minimum, 25th percentile, median, 75th percentile, maximum, and the difference between the first measurement and the most recent.

A series of episode summaries were then created, consisting of the statistical summaries at fixed time windows relative to the first measurement. Inclusion flowchart is shown in Figure s1. Univariate predictive performance for each summary variable for the training data is shown in Figure s2.

ML Model Implementation

Gradient boosting (GB) used the LightGBM implementation of the GB algorithm (LightGBM version 4.1.0), random forest (RF) and Logistic regression (LR) used sci-kit learn implementations (sci-kit learn version 1.3.2) and Bayesian networks (BN) used the Hugin Expert software (version 9.5, Hugin Expert A/S, Aalborg, Denmark).

Both GB and RF are ensemble learning method based on decision trees. However, GB seeks to reduce error mainly while reducing bias, while random forest seeks to reduce variance.

LR is a widely used traditional statistical technique for binary classification.

BN are graphical models based on directed acyclic graphs. Relationships between variables, which can be latent or observable, are encoded as conditional probability tables.

Model-specific Preprocessing

To avoid model-specific pitfalls and other issues, some of the models had additional preprocessing steps included in the machine learning pipeline, such as imputation, scaling, and other transformations such as fitting restricted cubic splines to allow potential non-linearities to be handled in linear models. For example, when regularization is used as part of a logistic regression fit, the final model is very sensitive to the scale of the data. By incorporating these steps in the pipeline, transformers are fitted only on the training data and the combined pipeline can be used directly with the test data without data leakage.

For GB and BN, no additional preprocessing was required. For RF missing values were replaced with extreme values to serve as an indicator of missingness. For LR missing values were imputed as column medians, values were scaled to have mean 0 and unit variance, and non-categorical variables were transformed using restricted cubic splines with 5 knots. GCS and the pain score were considered categorical.

Feature Impact Assessment

NEWS2 is a categorical score, with a fixed score between thresholds. The impact of a given measurement is therefore equal to the score given to its value.

The LR model used the restricted cubic splines technique, which uses a more flexible piecewise model. The impact is the effective weight of the spline components for each value, which is the equivalent of the logistic regression coefficient.

The BN model uses a similar technique, where each variable is decomposed into a set of Gaussian (normal) distributions. The effect of a single feature on the BN model can be

measured using the Bayes factor, which is the ratio of the posterior- to the prior odds ratio for a given hypothesis against a competing hypothesis, in this case 7-day mortality true/false. SHAP is a model-agnostic method of determining the relative contribution of each feature to a given prediction, and in this case is used for GB.

Linear models/EWS (NEWS2, DEPT, LR) have a fixed contribution for each unique feature value, while the contribution in the non-linear models (GB) depends on the value of other features.

BN Model Structure and Assumptions

Structure

The BN Model Structure is shown below. Red nodes are outcomes/diagnosis nodes, orange are “mapping nodes” and green are observed variables.

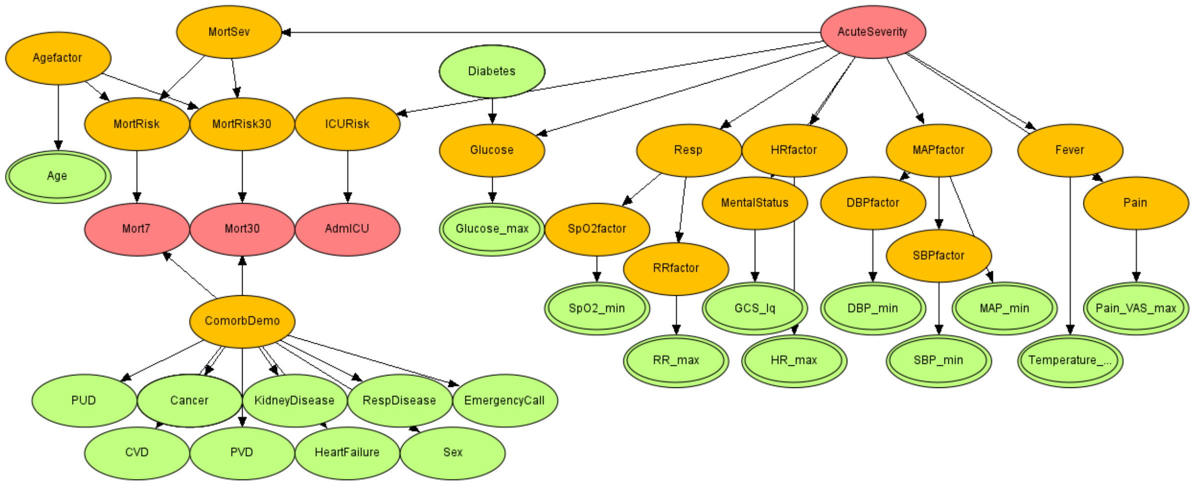

Use of discrete and continuous node types

The BN model uses both discrete and continuous nodes. Discrete nodes are represented by a set of discrete states e.g. yes/no, low/medium/high etc. The conditional probability table for a discrete node is defined for each node state, for each state or combination of states of the parent node(s). Continuous nodes do not have any states, and the conditional probability table is defined by assigning the mean and variance of a Gaussian (normal) distribution for each state or combination of states of the parent node(s). In the BN structure, continuous nodes are shown with a double outline.

Assumptions

Similar to previous work[2–4], acute severity was described using five states; no, mild, moderate, severe, and critical. Each state was associated with increasing mortality. Mapping nodes are then added to describe the probability distribution for each severity state across a set of Gaussian distributions spanning the range of physiological measurements. The Gaussian distributions themselves are fixed, and it is then the conditional probability tables of the mapping nodes that are learned. This results in a similar model to the use of restricted cubic splines in logistic regression. It was assumed that acute severity, age, sex, emergency call status, and comorbidity independently contribute to the mortality outcomes. Learning was restricted to the “mapping nodes” for the continuous variables and the observed discrete nodes i.e. mortality, ICU admission, comorbidities, sex, and emergency call status.

Results

For each model, performance was significantly higher for feature set 2 vs. feature set 1, and for feature set 3 vs. feature set 2. Exact variables used for each feature set are shown in

table s1. Predictive performance for the best summary statistics are included in table s2. Hyperparameter used within grid search for selected values in table s3, and training and test set performance for all models in table s4.

Subgroup analysis

The results of the subgroup analysis are shown in Figure s3 (diagnosis groups), Figure s4 (age groups) and Figure s5 (sex groups).

Patients were grouped according to the ICD-10 diagnosis chapter. Some differences were seen in various diagnosis subgroups, with lower performance seen for respiratory, digestive, endocrine/metabolic, infectious, genitourinary diagnoses compared to that for the combined patient population. For each diagnosis group, the performance advantage of the ML scores remained vs. the existing EWS.

Similarly, differences were observed across the six age strata, with a tendency to higher AUROC for younger patients. However, precision is influenced by the prevalence of the outcome, which increases significantly with age for both mortality outcomes.

Small differences were seen for the sex subgroups, with LR, GB and RF models tending to higher performance for male patients, and BN tending to higher performance for female patients.

Sensitivity analysis

Predictions should be available to paramedics as soon as possible during the care episode to have the most impact. We investigated model performance at a set of fixed cut-off times after the beginning of the episode, from 5 to 30 minutes, in each case only using data measured prior to that time. Performance of all models increased with increasing availability of data/data from an extended time window for both the AUROC and average precision metrics. AUROC and AUPRC are shown for each model at each time window in Figure s6.

When excluding patients who died on day 1, performance was lower for both the AUROC and average precision metrics for all models with the exception of NEWS2+Age for both mortality outcomes (now: 2-7 day mortality and 2-30 day mortality). No difference was observed for the ICU admission outcome. AUROC and AUPRC are shown, contrasting the sensitivity analysis and main analysis for each model and EWS, are shown in Figure s7.

Sensitivity to very short term mortality is shown in Figure s8.

Tables

Table 1 Descriptive statistics for train and test cohorts

Characteristic	Patients, No. (%)<br>Total cohort<br>(N = 219 323)	Train<br>(N = 175 458)	Test<br>(N = 43 865)
Age, median (IQR), y	69 (52-80)	69 (52-80)	69 (52-80)
Sex			
Female	104 699 (47.7)	83 759 (47.7)	20 940 (47.7)
Male	114 624 (52.3)	91 699 (52.3)	22 925 (52.3)
Called emergency number	119 992 (54.7)	95 993 (54.7)	23 999 (54.7)
Charlson comorbidity index, mean (sd), points	0.9 (1.3)	0.9 (1.3)	0.9 (1.3)
Acute severity, mean (sd), points			
NEWS2	3.2 (2.6)	3.2 (2.6)	3.2 (2.6)
DEPT	2.1 (1.0)	2.1 (1.0)	2.1 (1.0)
Admission			
Hospital	198 264 (90.4)	158 578 (90.4)	39 686 (90.5)
ICU admission	5044 (2.3)	4037 (2.3)	1007 (2.3)
Mortality, crude			
1d	4119 (1.9)	3310 (1.9)	809 (1.8)
7d	9334 (4.3)	7522 (4.3)	1822 (4.2)
30d	18650 (8.5)	14920 (8.5)	3730 (8.5)

Table 2 Predictive performance for the best summary statistics across all measurements within an episode (temporal variables) and for demographic and comorbidity variables (time invariant). The Feature set columns denote whether a variable was considered for use in a given feature set (1, 2, 3).

Variable	Summary	% measured	AUROC	1	2	3
Age	-	100.0	0.697		x	x
Sex	-	100.0	0.520			x
112 call (yes/no)	-	100.0	0.524			x
Comorbidities						
Charlson comorbidity index	-	70.9	0.633			x
Myocardial infarction	-	70.9	0.500			
Heart Failure	-	70.9	0.530			x
Peripheral vascular disease	-	70.9	0.511			x
Cerebrovascular disease	-	70.9	0.508			x
Respiratory disease	-	70.9	0.537			x
Peptic ulcer disease	-	70.9	0.503			x
Moderate Liver Disease	-	70.9	0.500			
Severe Liver Disease	-	70.9	0.500			
Diabetes (uncomplicated)	-	70.9	0.511			x
Diabetes (complicated)	-	70.9	0.504			x
Hemiplegia	-	70.9	0.500			
Kidney Disease	-	70.9	0.524			x
Cancer (other)	-	70.9	0.559			x
Hematological cancer	-	70.9	0.507			x
Metastatic cancer	-	70.9	0.511			x
HIV	-	70.9	0.500			
Heart rate↑	Max	92.9	0.656	x	x	x
SpO2↓	Min	92.0	0.747	x	x	x
Respiratory rate↑	Max	85.8	0.679	x	x	x
Glasgow coma scale↓	LQ	88.6	0.695	x	x	x
Systolic blood pressure↓	Min	90.5	0.654	x	x	x
Diastolic blood pressure↓	Min	90.5	0.644	x	x	x
Mean arterial pressure (calculated)↓	Min	88.7	0.652	x	x	x
Temperature↓	Median	41.8	0.470	x	x	x
Pain score (VAS)↓	Max	26.7	0.612	x	x	x
Blood glucose↓	Min	25.3	0.612	x	x	x

Table 3 Hyperparameter used within grid search, selected values

Model	Hyperparameter	Description	Values searched	Chosen
LR	C	Regularization strength	[1, 0.5, 0.1]	1
	L1 ratio	Ratio between LASSO and Ridge penalization.	[0, 0.1, 0.25, 0.5, 0.75, 0.9, 1]	0
GB	num_leaves	Max number of leaves in a tree	[3, 5, 8, 11, 14, 18]	14
	max_bin	Max number of discrete levels for each feature	[2, 4, 6, 8, 10, 15, 20, 25]	10
	learning_rate	Shrinkage rate	[0.005, 0.02, 0.05, 0.1, 0.2, 0.5]	0.2
	max_depth	Max depth of each tree	[2, 4, 5, 6, 8, 10]	10
	min_data_in_leaf	Minimum data points in one leaf	[20, 50, 100, 200, 500, 1000]	1000
	min_gain_to_split	Minimum performance gain to perform a split	[10, 20, 50]	10
	num_iterations	Number of boosting iterations (number of trees)	[20, 30, 50, 100, 200, 500, 1000]	500
	lambda_l1	L1 (LASSO) regularization strength	[0, 0.5, 1, 2, 10, 20]	2
	lambda_l2	L2 (Ridge) regularization strength	[0, 0.5, 1, 2, 10, 20]	0.5
RF	n_estimators	The number of trees in the forest	[100, 200, 500]	100
	max_depth	Max depth of each tree	[2, 4, 5, 6, 7, 8, 10]	10
	min_samples_leaf	Minimum data points in one leaf	[10, 50, 100, 200, 500, 1000]	10
	max_features	Number of features to consider when looking for the best split.	[5, 10, sqrt, log2, None]	sqrt
	class_weight	Weights associated with positive/negative class	[balanced, balanced_subsample, None]	None
	max_leaf_nodes	Max number of leaves in a tree	[5, 8, 11, 14, 20]	20

Table 4 Training and test set performance for all models. For each outcome, AUROC and AUPRC are measured.

Model | Feature Set	Outcome: 7-day mortality				Outcome: 30-day mortality				Outcome: ICU admission			
	Data: Train		Data: Test		Data: Train		Data: Test		Data: Train		Data: Test	
	AUROC	AUPRC	AUROC	AUPRC	AUROC	AUPRC	AUROC	AUPRC	AUROC	AUPRC	AUROC	AUPRC
GB | Set 1	0.859	0.416	0.853	0.418	0.796	0.373	0.786	0.362	0.753	0.134	0.747	0.134
GB | Set 2	0.881	0.443	0.877	0.442	0.835	0.412	0.829	0.403	0.707	0.097	0.695	0.099
GB | Set 3	0.896	0.464	0.891	0.460	0.862	0.449	0.858	0.440	0.701	0.090	0.691	0.092
LR | Set 1	0.824	0.331	0.822	0.338	0.759	0.316	0.754	0.313	0.735	0.143	0.727	0.149
LR | Set 2	0.860	0.370	0.861	0.376	0.818	0.370	0.816	0.367	0.697	0.100	0.693	0.105
LR | Set 3	0.875	0.384	0.875	0.387	0.846	0.405	0.844	0.402	0.695	0.091	0.691	0.094
BN | Set 1	0.808	0.312	0.809	0.331	0.737	0.301	0.734	0.306	0.744	0.144	0.730	0.137
BN | Set 2	0.846	0.334	0.849	0.346	0.799	0.343	0.797	0.346	0.719	0.094	0.706	0.091
BN | Set 3	0.852	0.328	0.857	0.345	0.813	0.351	0.814	0.355	0.715	0.087	0.705	0.087
RF | Set 1	0.831	0.385	0.830	0.403	0.760	0.344	0.756	0.341	0.738	0.120	0.731	0.123
RF | Set 2	0.858	0.417	0.858	0.425	0.801	0.378	0.799	0.376	0.712	0.098	0.704	0.100
RF | Set 3	0.870	0.431	0.872	0.438	0.829	0.401	0.829	0.399	0.696	0.094	0.688	0.096
NEWS + Age	0.786	0.174	0.791	0.176	0.770	0.254	0.771	0.251	0.680	0.068	0.667	0.068
DEPT + Age	0.811	0.165	0.815	0.167	0.769	0.237	0.769	0.238	0.684	0.060	0.666	0.058

Figures

Figure s1 Univariate performance and missingness for candidate variables. Upper panel: univariate AUROC for prediction of 7-day mortality. Lower panel: percentage of episodes where the variables were recorded. Summaries were included using data available at a set time after the first measurement. E.g. HR_5 includes summaries for heart rate measurements made in the first five minutes of each episode.

AUROC: Area Under the Receiver Operating Characteristics, Koen: sex, Call112_all: Emergency number call, Charlson_original: Charlson Comorbidity Index score.

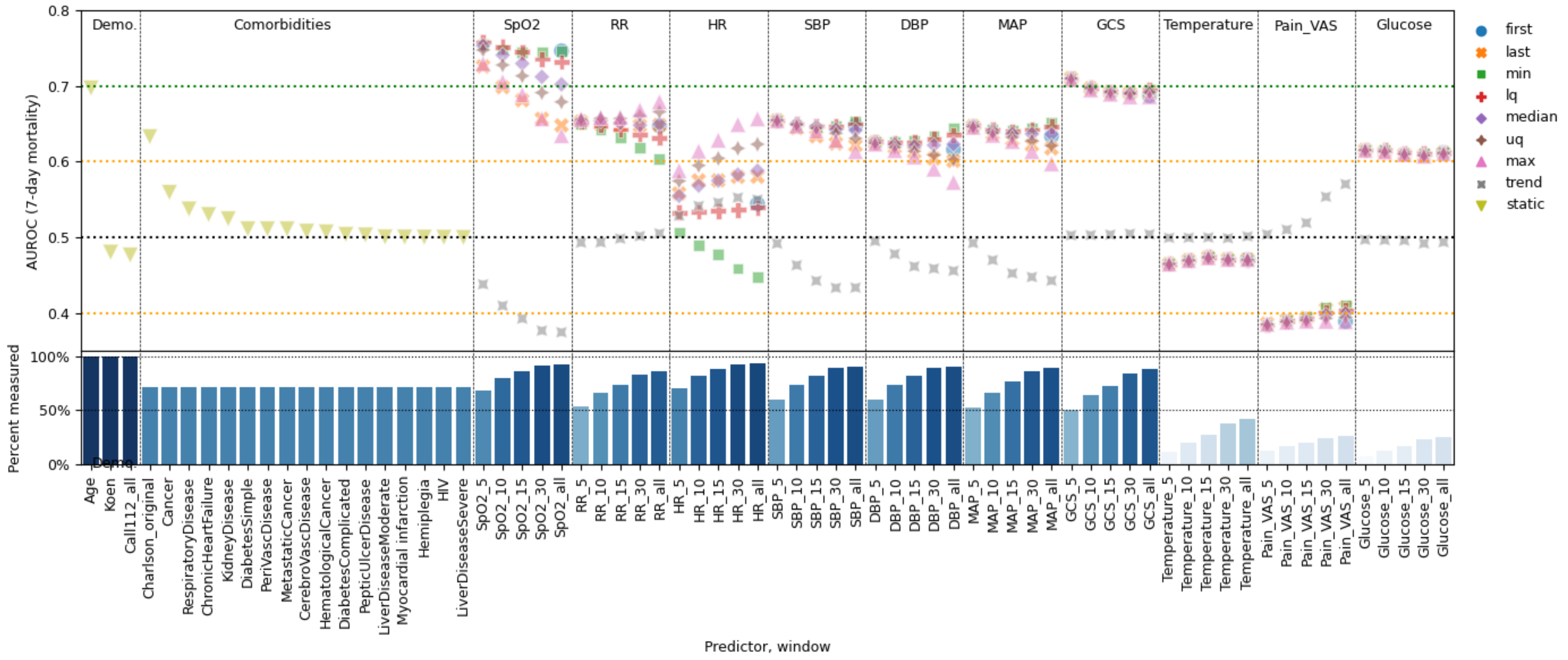

Figure s2 Inclusion flowchart

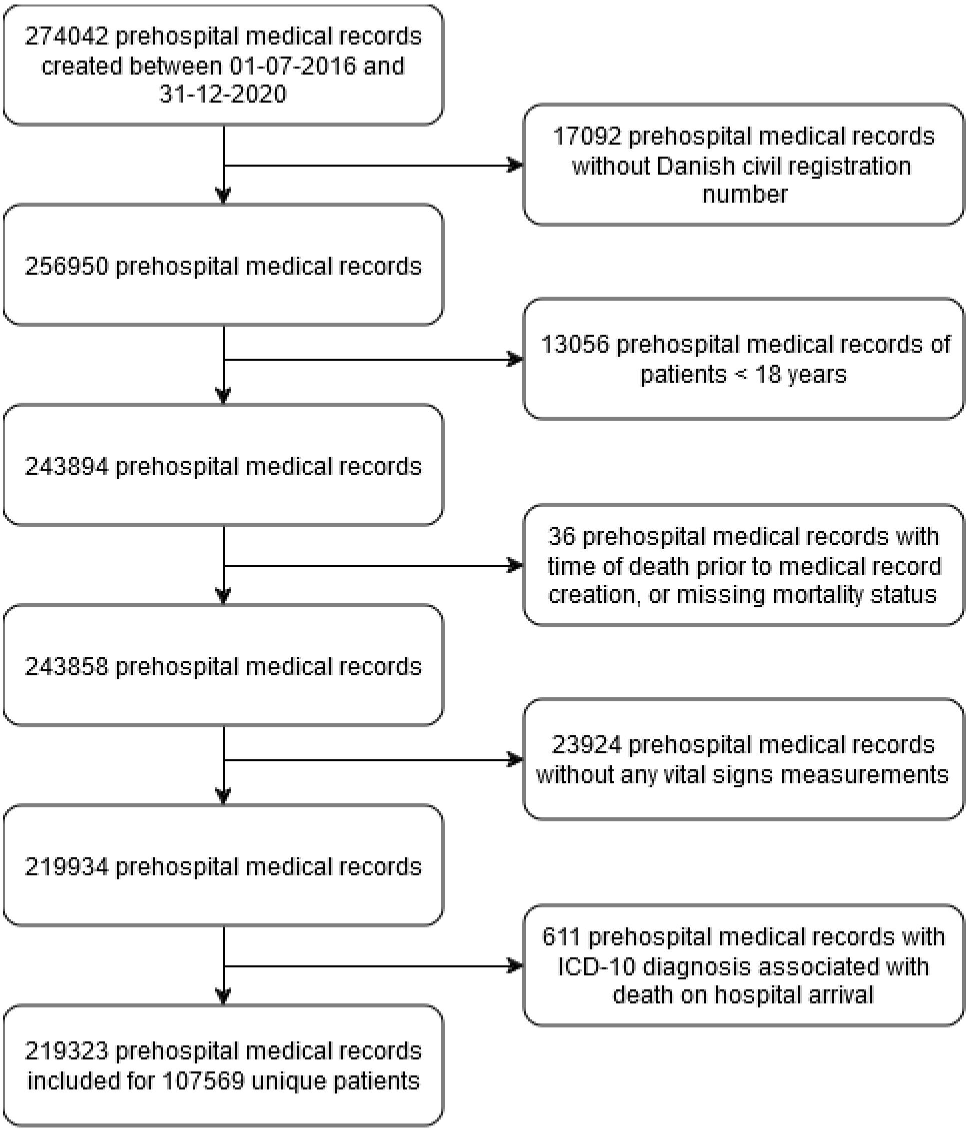

Figure s3 Predictive performance in diagnosis chapters. AUROC (95% CI) for 7-day mortality (left), 30-day mortality (centre) and ICU admission (right) for patients stratified by initial ICD-10 chapter.

AUROC: Area Under the Receiver Operating Characteristics, CI: Confidence interval, ICD-10: International Statistical Classification of Diseases and Related Health Problems, M7: 7-day mortality, M30: 30-day mortality, ICU: Intensive care unit admission, LR: Logistic Regression, GB: Gradient-boosting, RF: Random Forest, BN: Bayesian Network, NEWS2: National Early Warning Score 2, DEPT: Danish Emergency Process Triage.

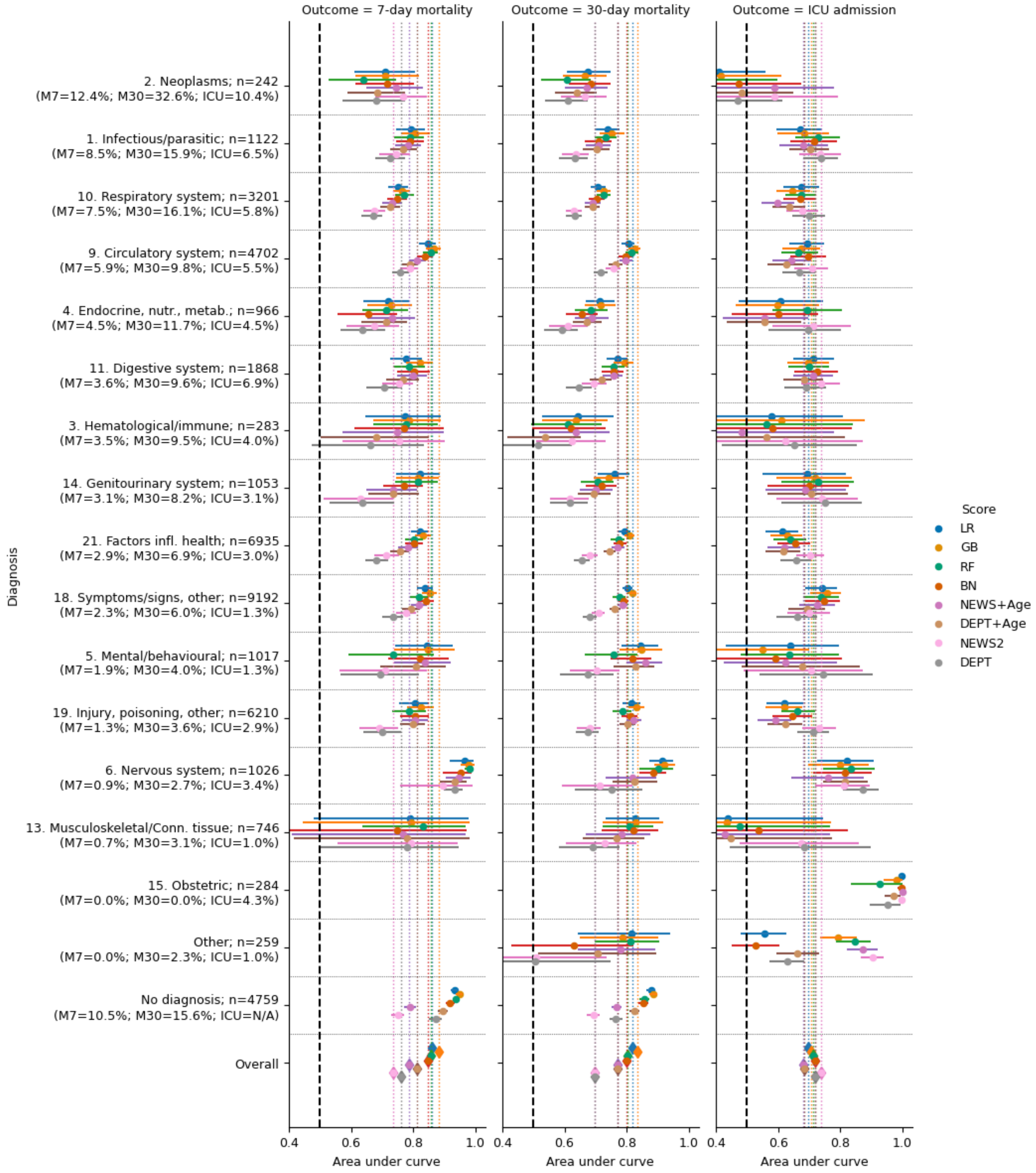

Figure s4 Predictive performance in diagnosis chapters. Average precision (95% CI) for 7-day mortality (left), 30-day mortality (centre) and ICU admission (right) for patients stratified by initial ICD-10 chapter.

CI: Confidence interval, ICD-10: International Statistical Classification of Diseases and Related Health Problems, M7: 7-day mortality, M30: 30-day mortality, ICU: Intensive care unit admission, LR: Logistic Regression, GB: Gradient-boosting, RF: Random Forest, BN: Bayesian Network, NEWS2: National Early Warning Score 2, DEPT: Danish Emergency Process Triage.

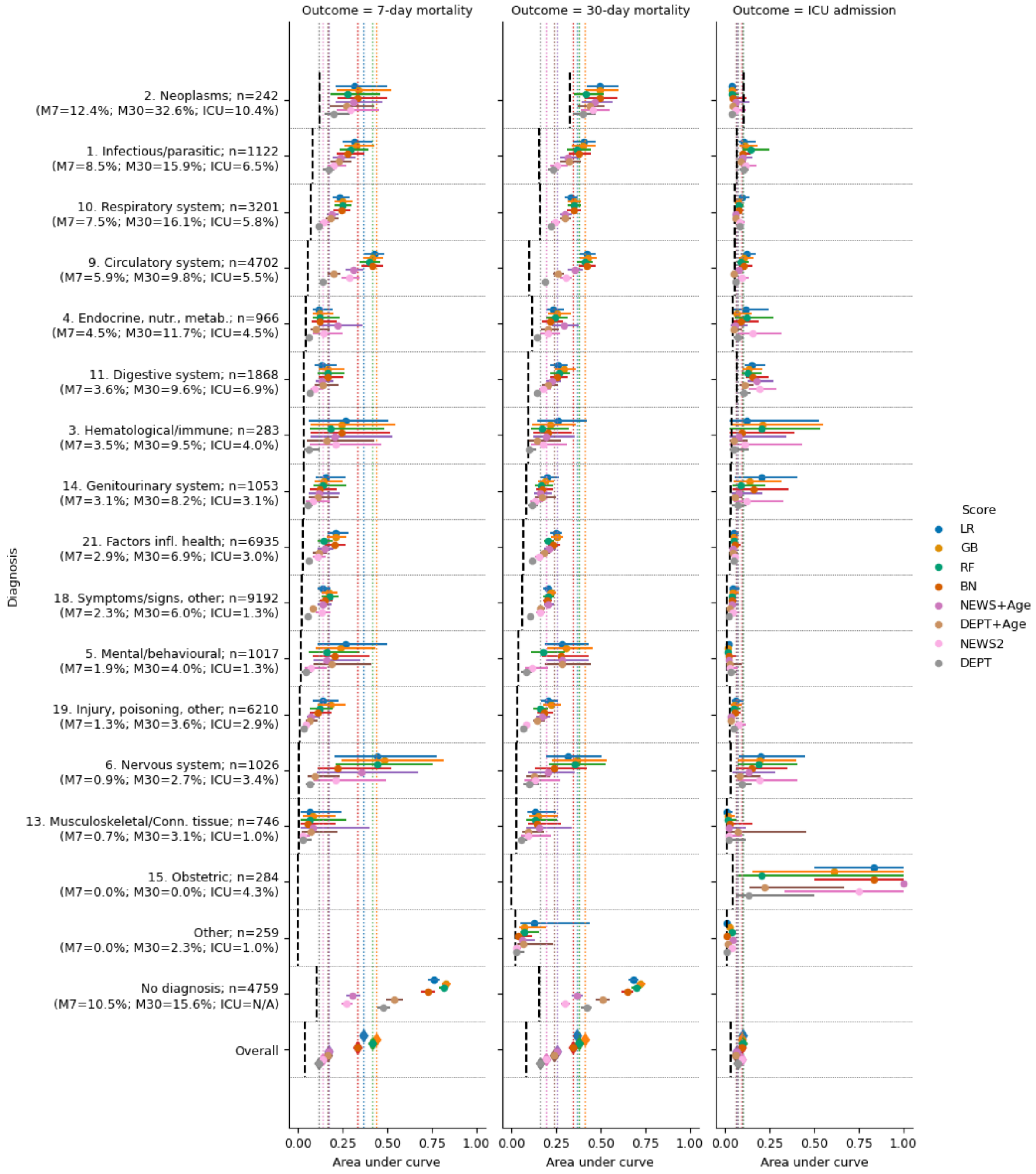

Figure s5 Upper panel: AUROC (95% CI) for 7-day mortality (left), 30-day mortality (centre) and ICU admission (right) for patients stratified by age group. Lower panel: Average precision (95% CI) for 7-day mortality (left), 30-day mortality (centre) and ICU admission (right) for patients stratified by age group.

AUROC: Area Under the Receiver Operating Characteristics, CI: Confidence interval, ICU: Intensive care unit admission, LR: Logistic Regression, GB: Gradient-boosting, RF: Random Forest, BN: Bayesian Network, NEWS2: National Early Warning Score 2, DEPT: Danish Emergency Process Triage.

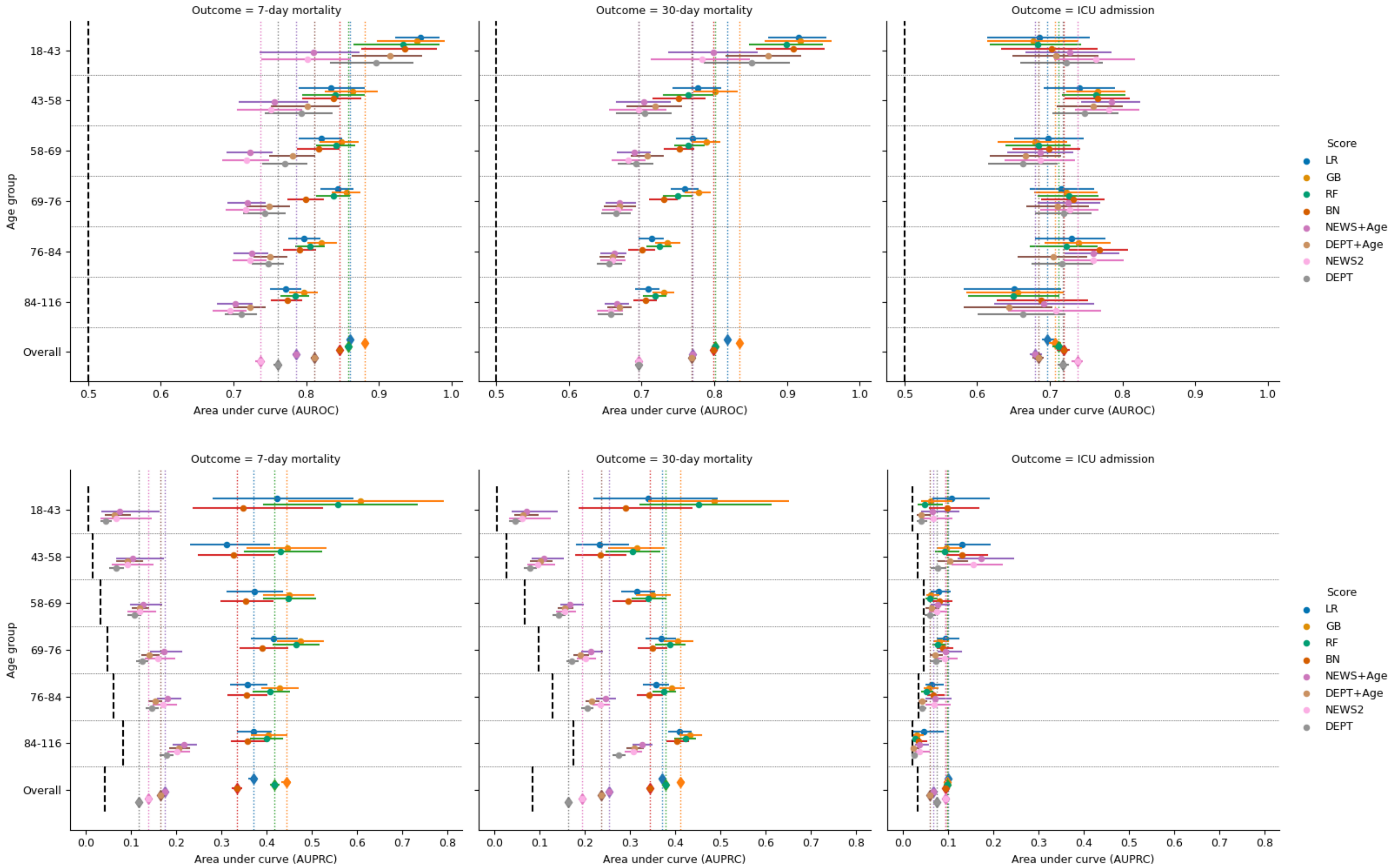

Figure s6 Upper panel: AUROC (95% CI) for 7-day mortality (left), 30-day mortality (centre) and ICU admission (right) for patients stratified by sex. Lower panel: Average precision (95% CI) for 7-day mortality (left), 30-day mortality (centre) and ICU admission (right) for patients stratified by sex

AUROC: Area Under the Receiver Operating Characteristics, CI: Confidence interval, ICU: Intensive care unit admission, LR: Logistic Regression, GB: Gradient-boosting, RF: Random Forest, BN: Bayesian Network, NEWS2: National Early Warning Score 2, DEPT: Danish Emergency Process Triage.

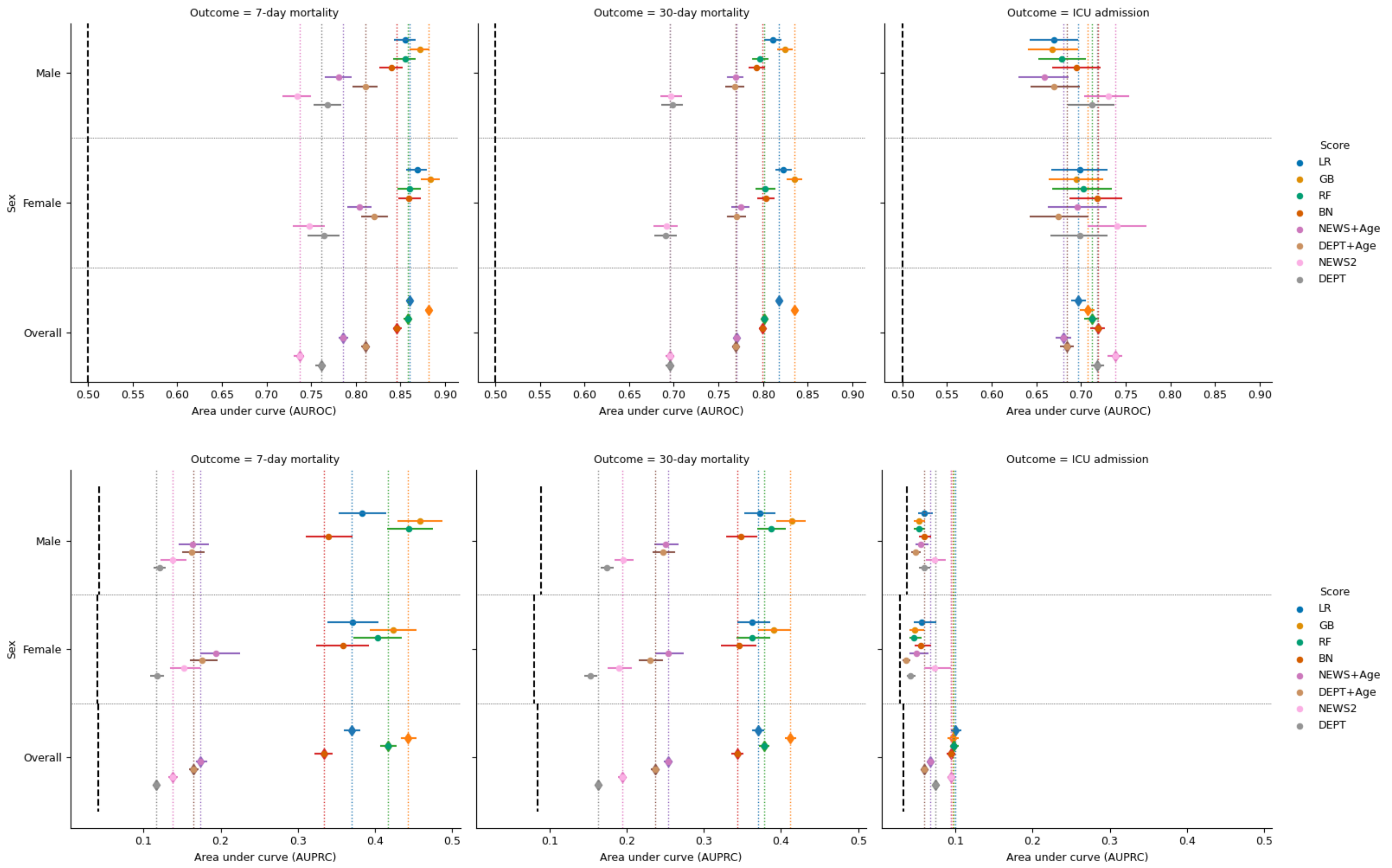

Figure s7 Impact of time horizon on model predictive ability. The models were provided with data summaries based on measurements up until the specified number of minutes after the episode start. Upper panel: AUROC. Lower Panel: AUPRC for 7-day mortality (left), 30-day mortality (centre) and ICU admission (right).

AUROC: Area Under the Receiver Operating Characteristics, CI: Confidence interval, ICU: Intensive care unit admission, LR: Logistic Regression, GB: Gradient-boosting, RF: Random Forest, BN: Bayesian Network, NEWS2: National Early Warning Score 2, DEPT: Danish Emergency Process Triage.

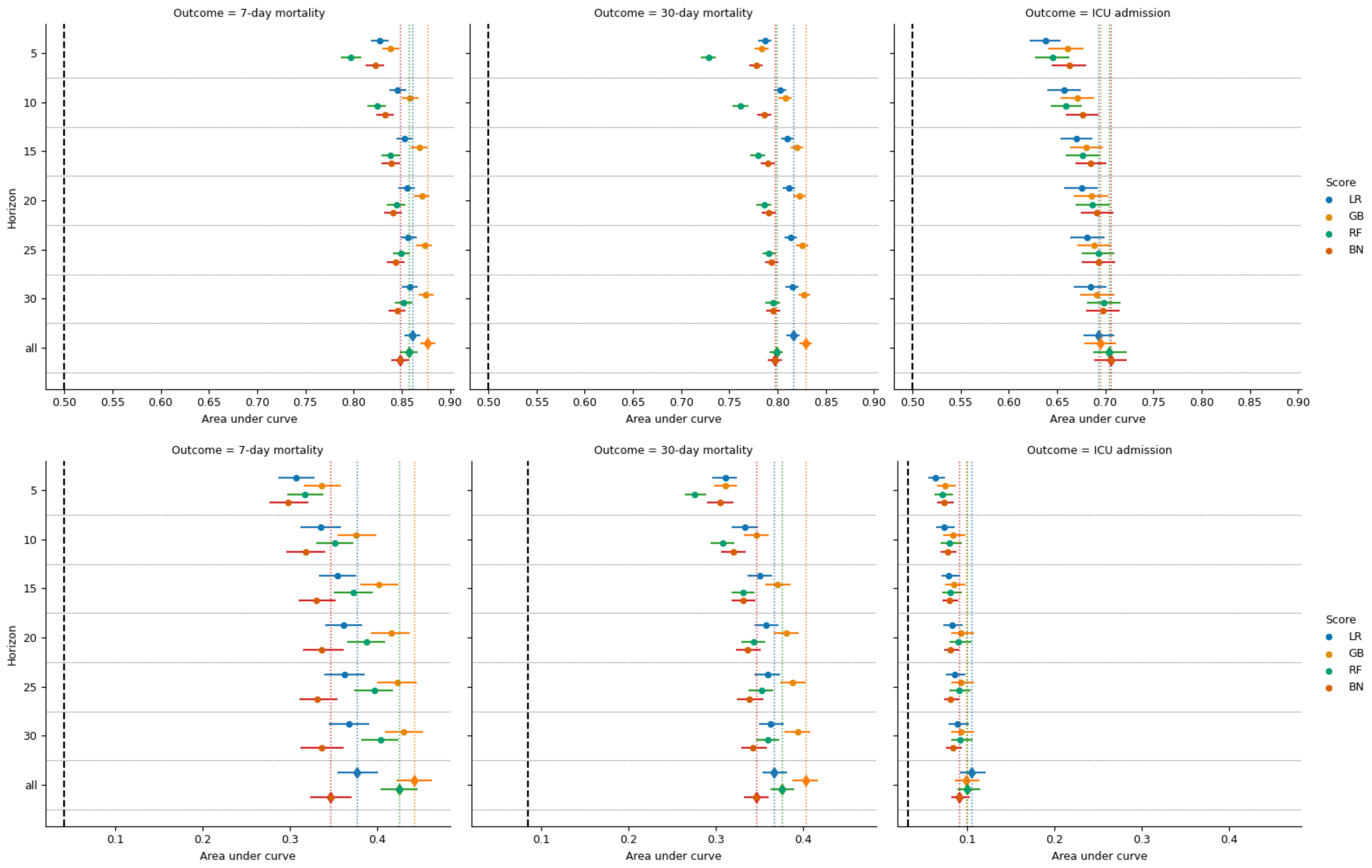

Figure s8 Sensitivity to very short term mortality. AUROC (upper panels) and AUPRC (lower panels) (95% CI) are shown for each model, for 7-day mortality (left), 30-day mortality (centre) and ICU admission (right). For each model, performance is shown for all patients (diamonds) and for the cohort excluding all patients who died on day 1 (crosses).

AUROC: Area Under the Receiver Operating Characteristics, CI: Confidence interval, ICU: Intensive care unit admission, LR: Logistic Regression, GB: Gradient-boosting, RF: Random Forest, BN: Bayesian Network, NEWS2: National Early Warning Score 2, DEPT: Danish Emergency Process Triage.

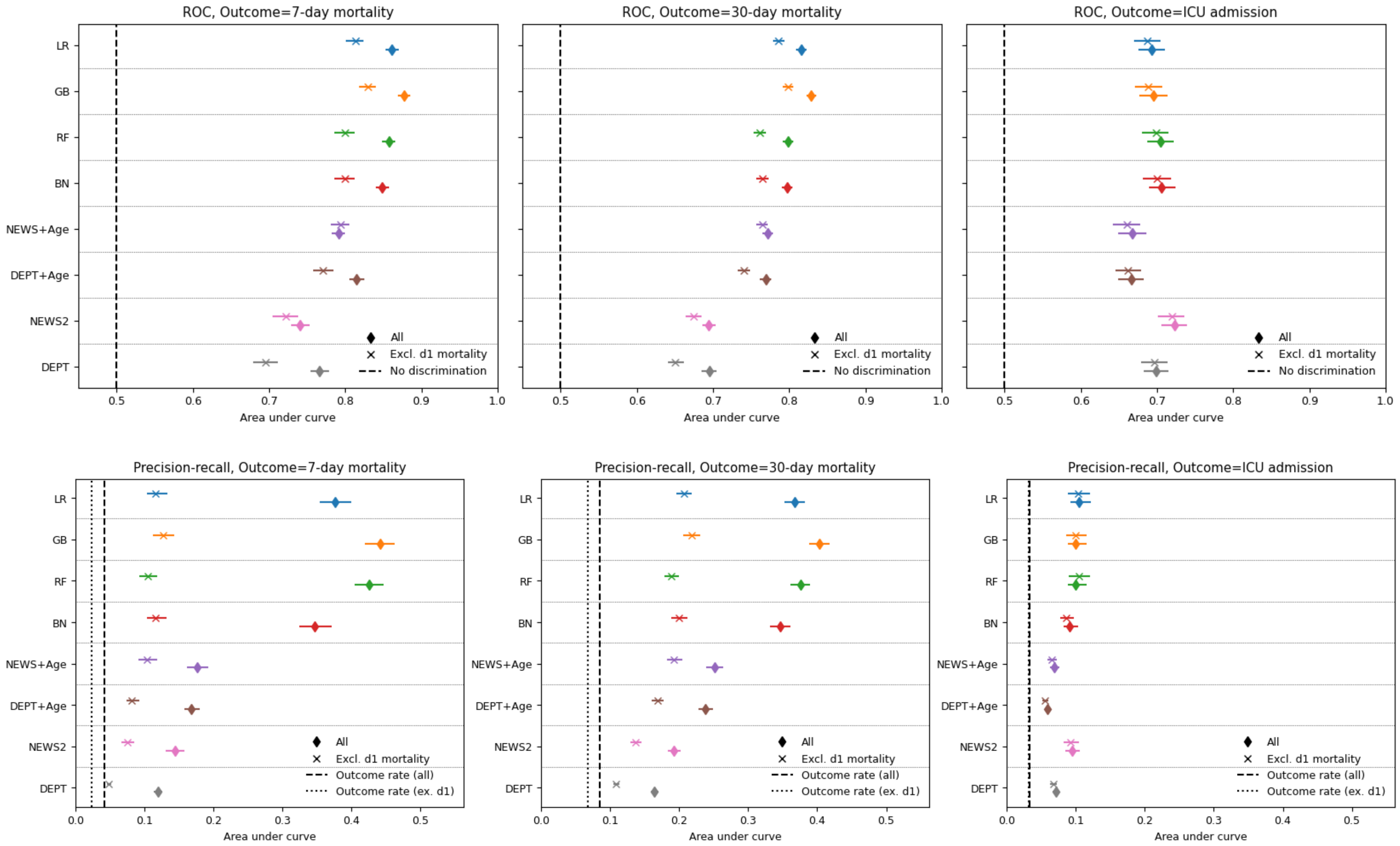

References

1. Lindskou TA, Ward LM, Søvsø MB, Mogensen ML, Christensen EF. Prehospital Early Warning Scores to Predict Mortality in Patients Using Ambulances. *JAMA Netw Open*. 2023;6(8):e2328128. doi:10.1001/jamanetworkopen.2023.28128

2. Ward L, Paul M, Andreassen S. Automatic Learning of mortality in a CPN model of the Systemic Inflammatory Response Syndrome. *Math Biosci*. 2017;284:12-20. doi:http://dx.doi.org/10.1016/j.mbs.2016.11.004

3. Cilloniz C, Ward L, Mogensen ML, et al. Machine-Learning Model for Mortality Prediction in Patients with Community-acquired Pneumonia: Development and validation study. *Chest*. Published online July 2022. doi:10.1016/j.chest.2022.07.005

4. Valik JK, Ward L, Tanushi H, et al. Predicting sepsis onset using a machine learned causal probabilistic network algorithm based on electronic health records data. *Sci Rep*. 2023;13(1):11760. doi:10.1038/s41598-023-38858-4
